# Transcriptome Analysis of Poplar Under Salt Stress and Over-Expression of Transcription Factor *NAC57* Gene Confers Salt Tolerance in Transgenic *Arabidopsis*

**DOI:** 10.3389/fpls.2018.01121

**Published:** 2018-09-04

**Authors:** Wenjing Yao, Kai Zhao, Zihan Cheng, Xiyan Li, Boru Zhou, Tingbo Jiang

**Affiliations:** ^1^State Key Laboratory of Tree Genetics and Breeding, Northeast Forestry University, Harbin, China; ^2^Northeast Institute of Geography and Agroecology, Chinese Academy of Sciences, Harbin, China

**Keywords:** *Populus alba* × *Populus glandulosa*, NAC, transcription factor, salt stress, ROS

## Abstract

NAC domain genes belong to a large plant-specific transcription factor family, which is well-known to be associated with multiple stress responses and plant developmental processes. In this study, we screened differentially expressed genes (DEGs) and detected mRNA abundance of NAC family by RNA-Seq in the poplar leaves under salt stress condition. A total of 276 up-regulated DEGs and 159 down-regulated DEGs were identified to be shared in *Populus alba* × *Populus glandulosa* and *Populus simonii* × *Populus nigra*. Among 170 NAC members, *NAC57* gene was significantly up-regulated in response to salt stress in the two species. Tissue-specific and salt-responsive analyses indicated the expression pattern of *NAC57* gene was spatial and temporal in poplar under salt stress. Particle bombardment results showed subcellular localization of NAC57 was not solely nucleus-targeted. Full-length cDNA sequence of the *NAC57* gene was cloned from *P. alba* × *P. glandulosa* and transformed into *Arabidopsis thaliana*. Under salt stress, transgenic *Arabidopsis* overexpressing *NAC57* showed higher seed germination rate, root length, and fresh weight than wild type plants. In addition, the transgenic plants displayed higher superoxide dismutase activity and peroxidase activity, and lower malondialdehyde content and relative electrical conductivity than the wild type under salt stress condition. Furthermore, histochemical staining indicated reactive oxygen species accumulation was lower in the transgenic plants than that in the wild type under salt stress. All the results indicated that the *NAC57* gene plays an important role in salt stress responses.

## Introduction

Salinity is one of the most challenging abiotic stresses that threaten plant growth and development. Recent studies on plant abiotic stress responses were primarily focused on TFs regulation, ABA receptor identification, signal transduction and phytohormone mediation (Qin et al., 2011). TFs act as master regulators by activating or repressing a large number of functional genes through binding to stress-related *cis*-elements in the promoters of downstream target genes (Gujjar et al., 2014; Liu et al., 2014). Understanding regulatory mechanism of TFs will facilitate deciphering complex signaling networks in plant stress responses (Shao et al., 2015). The networks of TFs with roles in environmental stress responses are unraveling and hundreds of genes encoding TFs participated in abiotic stress responses have been identified in recent years (Chen and Zhu, 2004).

Plants adapt to environmental changes by dramatically altering gene expression level or pattern, and these transcriptional changes lead to stress tolerance (Hirayama and Shinozaki, 2010; Muchate et al., 2016). The stress-responsive genes can be broadly classed into three categories: functional genes, regulatory genes and genes with unknown function (Bhatnagarmathur et al., 2008). Among regulatory genes, stress-related TFs, such as ERFs, WRKYs, and bZIPs, were identified to be induced or repressed by a range of environmental stresses, and overexpression of a few TFs can enhance stress tolerance of transgenic plants (Singh et al., 2002). For example, *ERF76* gene was induced significantly in the leaves, roots and stems of *Populus simonii* × *Populus nigra* under salt stress (Yao et al., 2016a). Overexpression of the *ERF76* gene enhanced salt stress tolerance of transgenic poplar by regulating the expression level of 375 functional DEGs including 268 up-regulated genes and 107 down-regulated genes (Yao et al., 2016b). A wheat WRKY gene, *TaWRKY44*, was induced by multiple treatments including PEG6000, NaCl, low temperature, ABA, GA, and H_2_O_2_, and overexpression of this gene in transgenic tobacco conferred drought and salt tolerance via up-regulating some reactive oxygen species (ROS)-related genes and stress-responsive genes under osmotic stresses conditions (Wang et al., 2015). *ABP9*, a bZIP gene from maize, enhanced tolerance to salt and drought in transgenic cotton by altering physiological and biochemical processes and regulating stress-related gene expression (Wang et al., 2017). Similar studies have been productive in recent years for identifying TFs involved in plant stress responses.

As one of the largest TF families in plants, the NAC family not only plays vital roles in regulating plant growth and development, but also participates in complex signaling networks during plant stress responses (Nakashima et al., 2012; Puranik et al., 2012). A large spectrum of NAC members from multiple species have been identified in recent years. A NAC gene from rice, *ONAC022*, was induced by drought, high salinity and ABA, and improved drought and salt stress tolerance through modulating an ABA-mediated pathway in transgenic plants (Hong et al., 2016). *TsNAC1* from halophyte *Thellungiella halophila* played a positive role in abiotic stress resistance, especially in salt stress tolerance, in both *T. halophila* and *Arabidopsis* by targeting positive regulators of ion transportation (Liu et al., 2017). Meanwhile, *ONAC095*, a NAC gene from rice, played negative roles in drought and cold stress tolerance (Huang et al., 2016). Functional characterization of NAC genes can help to understanding regulatory mechanism of TF genes in plant stress responses and generating stress tolerant transgenic plants by overexpression techniques (Shao et al., 2015).

In this study, we first screened DEGs and detected expression change of NAC family in the leaves of *P. alba* × *P. glandulosa* and *P. simonii* × *P. nigra* under salt stress by RNA-Seq. Then we profiled relative expression level of *NAC57* gene in different tissues at different developmental stages and investigated expression pattern of *NAC57* gene in the leaves of *P. alba* × *P. glandulosa* under salt stress by RT-qPCR. Furthermore, we constructed NAC57-GFP fusion and confirmed subcellular localization of NAC57 protein by particle bombardment. Moreover, we cloned full-length cDNA of *NAC57* gene from *P. alba* × *P. glandulosa* and transformed the gene into *Arabidopsis thaliana* by floral dip method, and obtained 13 transgenic *Arabidopsis* lines overexpressing *NAC57* gene, and conducted morphological measurement, physiological characterization and histochemical analysis of transgenic plants under salt stress. All the results indicated that the *NAC57* gene is tissue-specific, salt-inducible and its overexpression enhances salt tolerance in transgenic *Arabidopsis*.

## Materials and Methods

### Plant Culture and Salt Stress Treatment

The twigs of *P. alba* × *P. glandulosa* were hydroponics cultured at room temperature with 16/8 h light/dark cycles and 70% relative humidity for 2 months. The seedlings with new roots and leaves were challenged with 150 mM NaCl solution and water as control for 0, 6, 12, 24, and 48 h. The leaves at 0, 6, 12, 24, and 48 h of each treatment were frozen in liquid nitrogen and stored at -80°C for salt-responsive analysis. The leaves, stems, roots and cambiums under control condition were harvested for tissue-specific analysis. For each treatment, three biological replicates were prepared for each sample from ten seedlings, respectively.

Surface sterilization of *Arabidopsis* seeds was conducted as following: add 2 ml 20% (v/v) bleach solution to 2 ml microcentrifuge tube with about 20 μl seeds. After shaking for 15 min, centrifuge and discard the bleach solution. Then wash the seeds with sterile water for 4 times. The sterilized seeds were sowed on horizontal plates containing MS medium (pH 5.8–6.0) and placed at 4°C in the dark for 2 days. Then put the plates in 24°C, 16/8 h light/dark cycles condition for seedling (Yao et al., 2017a).

### Expression Analysis of DEGs and *NAC* Family Genes

*P. alba* × *P. glandulosa* seedlings with new roots and leaves were divided to two groups and subjected to 150 mM NaCl and water as control for 24 h, respectively. Each group contains three biological replicates and each biological replicate contains ten seedlings. The secondary leaf samples from six biological groups were sent to GENEWIZ company^1^ for RNA-Seq with Illumina HiSeq 2500 platform (All relevant RNA-Seq data generated and analyzed for this study are included in the **Supplementary Data Sheets S1, S2**, respectively. The raw RNASeq reads (Fastqc) supporting the conclusions of this manuscript will be made available by the corresponding author to any qualified researcher). The mRNA abundance of poplar genes were quantified as FPKM (fragments per kilo bases per million reads, **Supplementary Data Sheet S1**). The DEGs were determined by edgeR algorithm (corrected *p*-value ≤ 0.05) in Pop’s Pipes software: Poplar Gene Expression Data Analysis Pipelines^2^. The number of comparisons and default minimum value were set as 1 (Li et al., 2014). The hierarchical clustering of the DEGs was conducted using Gene Cluster 3.0 software.

We filtered NAC family members whose FPKM ≥ 4 in at least one biological experiment, and fold change (FC) of FPKM was applied to profile the expression trend of salt responsive NAC genes (Yao et al., 2017b).

### Expression Analysis of *NAC57* Gene

Total RNA was extracted from *P. alba* × *P. glandulosa* using Column Plant RNAout Kit (Tiandz, Beijing, China). The reverse transcript first-strand cDNA was synthesized using PrimeScript^TM^ RT reagent kit with gDNA Eraser (Takara, Dalian, China).

The RT-qPCR was performed using SYBR Premix Ex Taq^TM^ (Takara, Dalian, China) with above cDNA as template. Relative expression level of genes was calculated following 2^-ΔΔCt^ method (Livak and Schmittgen, 2001). The data was normalized using poplar actin gene, and the information of related primer pairs (ACT, EF, and NAC57-1) was listed in **Supplementary Table S1**. Three technical replications were conducted for each biological experiment. Mean values represent relative expression level, and error bars standard for deviation (±SD).

### Phylogenetic Tree Construction of the NACs

The deduced amino acid sequences of NAC family from *Populus trichocarpa* and *A. thaliana* was derived from PlantTFDB^3^. The information of NAC*57* gene from *P. alba* × *P. glandulosa* and a few NAC homologous genes from other species was derived from Phytozome 12^4^. Sequence alignment was conducted by Clustal W and phylogenetic tree was constructed by MEGA 6.0 using Neighbor-Joining method.

### Subcellular Localization of NAC57 Protein

To determine subcellular localization of NAC57 protein, the coding region of *NAC57* gene without stop codon (915 bp) was cloned and fused into pBI121 vector between Xba**I** and Spe**I** restriction enzyme sites using NAC57-2 primer pair (**Supplementary Table S1**), constructing NAC57-GFP fusion under the control of CaMV35S promoter. The constructed vector 35S::NAC57-GFP and 35S::GFP vector as control were transformed to onion epidermal cells by particle bombardment, and transient expression of fusion gene with GFP fluorescence signal was detected by fluorescence microscopy (LSM 700, Zeiss, Germany).

### Generation of Transgenic *Arabidopsis* Plants

The 996 bp transcript of *NAC57* gene containing 918 bp full-length cDNA was obtained by RT-PCR with a pair of specific primers NAC57-3 (**Supplementary Table S1**). The gene was inserted into pBI121 vector replacing GUS gene driven by CaMV35S promoter with XbaI and SacI restriction enzyme sites. The recombinant vector was transformed into EHA105 by electroporation system (Eppendorf 2510). Agrobacterium-mediated transformation of *Arabidopsis* was following the protocols of floral dip method (Zhang et al., 2006). Transgenic *Arabidopsis* plants were selected on MS medium with 50 mg/L kanamycin.

### Salt Stress Test of the Transgenic *Arabidopsis*

To test salt tolerance of transgenic plants over-expressing *NAC57*, we conducted morphological measurement, physiological characterization and histochemical detection of T2 transgenic *Arabidopsis* plants according to the methods described in previous study (Yao et al., 2016a).

## Results

### Differentially Expressed Genes and *NAC* Genes

**FIGURE 1:**
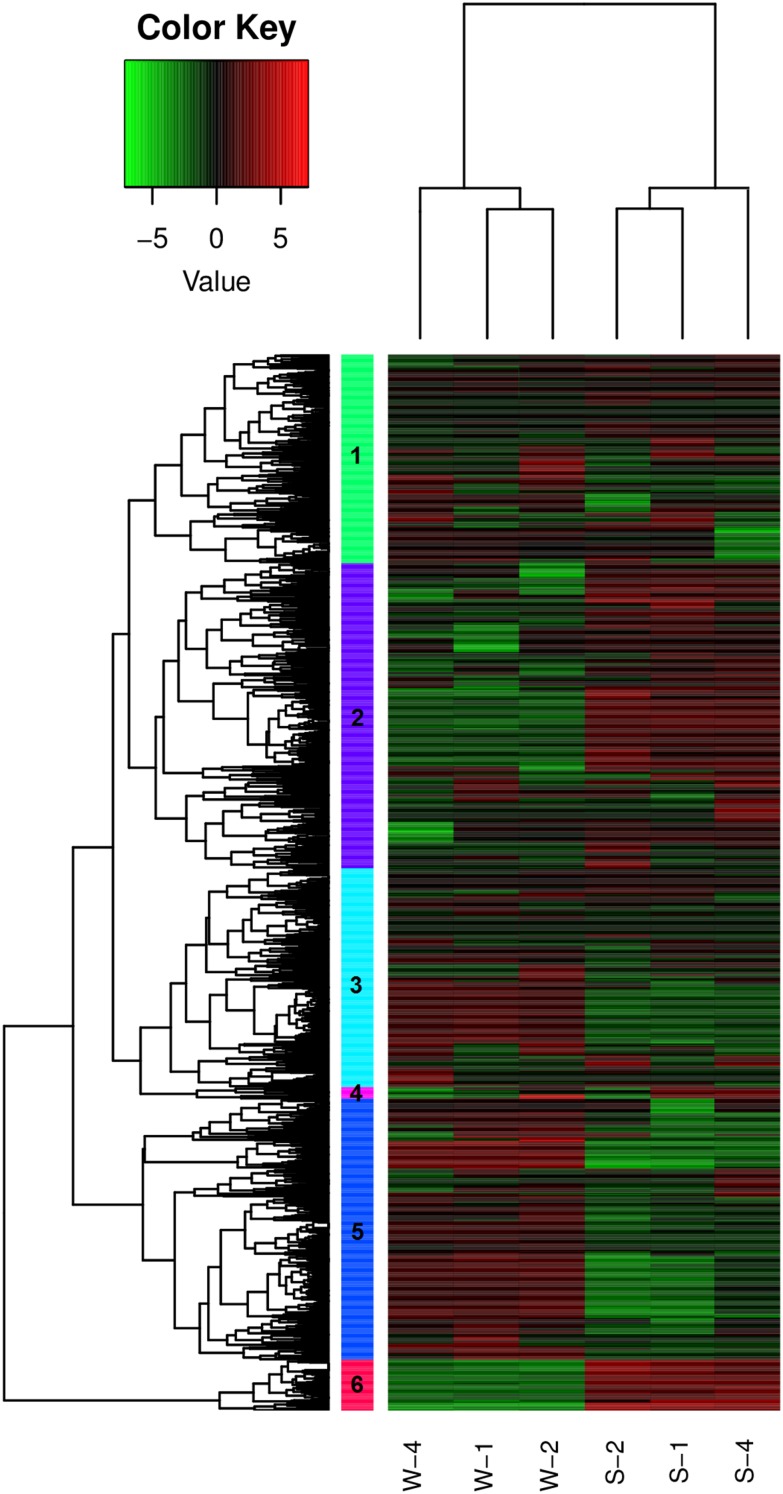
Heatmap of DEGs in leaves of *Populus alba* × *Populus glandulosa* based on RNA-Seq. The expression levels are quantified as log_2_ FPKM. Red and green colors indicate up- and down-regulated, respectively. The colored vertical bars denote different gene clusters. Three biological repicates S1, S2, and S4 were treated with 150 mM NaCl for 24 h, and sample W1, W2, and W4 were three control biological repicates.

The DEGs in *P. alba* × *P. glandulosa* under salt stress were profiled by RNA-Seq (**Supplementary Data Sheet S2**). A total of 65535 *Populus* genes were detected in the RNA-Seq data, and as many as 2198 DEGs (*FC* ≥ 2, corrected *p*-value ≤ 0.05) including 901 up-regulated genes and 1297 down-regulated genes (**Supplementary Data Sheet S2**) were identified based on Pop’s Pipes software (Li et al., 2014). According to hierarchical clustering of FPKM, these DEGs could be classified into six clusters in the heatmap under salt stress condition (**Figure 1**). Clusters 2 and 6 are mainly up-regulated genes in response to salt stress. In contrast, clusters 3 and 5 are mainly down-regulated genes, and DEGs in cluster 1 are mixed of up- and down-regulated genes (**Figure 1**). Based on our previous RNA-Seq data (Yao et al., 2017b), we also compared the DEGs and NAC family members in the leaves under salt stress between *P. alba* × *P. glandulosa* and *P. simonii* × *P. nigra*. The results indicated there are a total of 435 shared DEGs, including 276 up-regulated genes and 159 down-regulated genes (**Supplementary Data Sheet S2**). According to enriched GO terms, these DEGs are mainly associated with multiple biological processes such as metabolic process, biological regulation, single-organism process, localization, cellular process, developmental process, response to stimulus, etc. The function annotation of the shared DEGs was listed in **Supplementary Data Sheet S2**.

To understand expression patterns of NAC family members in *P. alba* × *P. glandulosa*, FPKM value of 170 NAC family genes was derived from the RNA-Seq data. A total of 76 salt-responsive NAC members were identified, including 54 up-regulated and 22 down-regulated genes. As many as 61 NAC genes were shared in *P. alba* × *P. glandulosa* and *P. simonii* × *P. nigra*. Among them, *NAC57* gene was significantly induced in both species by salt stress.

### Temporal and Spatial Expression Pattern of the *NAC57* Gene

**FIGURE 2:**
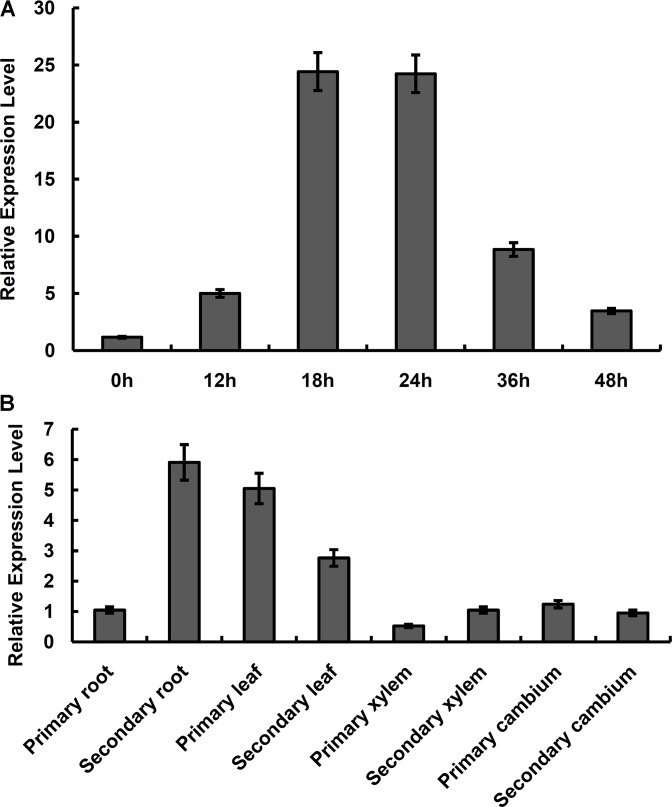
Relative expression level of *NAC57* gene in *P. alba* × *P. glandulosa*. **(A)** Relative expression levels of *NAC57* gene in leaves of *P. alba* × *P. glandulosa* under salt stress for 0–48 h; **(B)** Relative expression levels of *NAC57* gene in different tissues of *P. alba* × *P. glandulosa*. Mean values and deviations were calculated from three independent biological experiments.

The RT-qPCR results indicated that relative expression level of *NAC57* gene in the leaves of *P. alba* × *P. glandulosa* changed continuously under salt stress condition across treatment time course. As shown in **Figure 2A**, the relative expression level of *NAC57* gene increased during the 0–24 h period, reaching peak at 24 h time point and decreased during 24–48 h period. These results indicated that the *NAC57* gene was salt-inducible and the expression pattern of *NAC57* displayed an increase and then decrease trend during 0–48 h treatment.

We also conducted tissue-specific analysis of the *NAC57* gene in *P. alba* × *P. glandulosa* by RT-qPCR. The results indicated the expression of *NAC57* gene varied in different tissues (**Figure 2B**). The relative expression level of *NAC57* was highest in secondary roots and lowest in primary xylem, and the highest expression level was 11.3 times higher than the lowest. The mRNA abundance of *NAC57* also varied at different growth stages. For example, the relative expression level in primary leaves was higher than that in secondary leaves, while it was higher in secondary roots than that in principal roots (**Figure 2B**).

### Phylogenetic Analysis of NAC Transcription Factors

**FIGURE 3:**
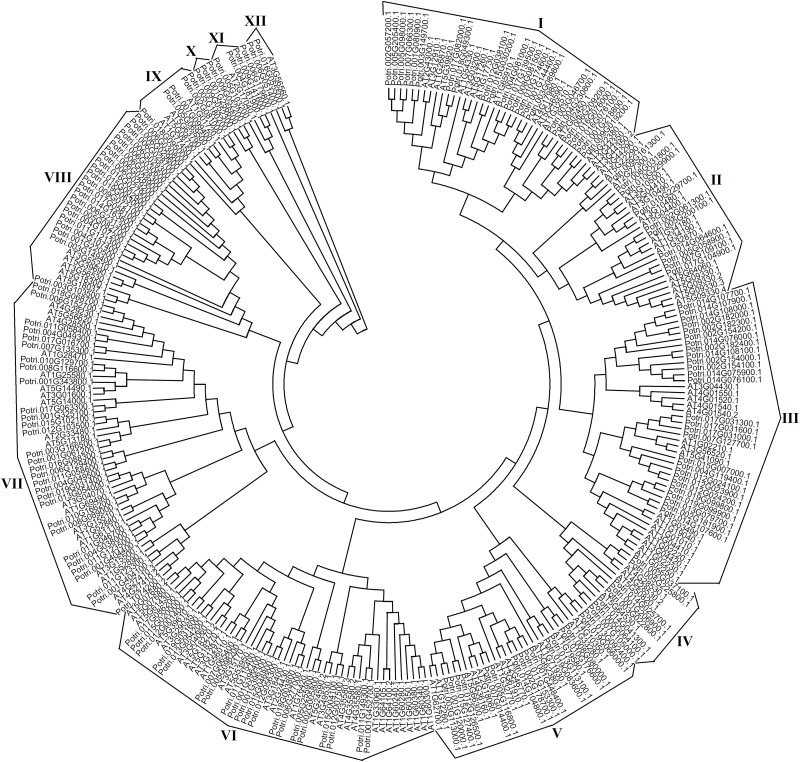
Phylogenetic tree of NAC family from *Populus trichocarpa* and *Arabidopsis thaliana* constructed by MEGA 6.0 with Neighbor-Joining method.

To examine evolutionary relationship of NAC TF families between *Populus* and *Arabidopsis*, a phylogenetic tree containing 170 NAC genes with distinct ORFs from *Populus trichocarpa* and 124 from *Arabidopsis thaliana* was constructed with their deduced protein sequences. The NAC proteins were divergent distinctly between the two species (**Figure 3**). The NAC gene (Potri006G179800) formed an individual clade and the other 169 poplar NAC proteins were clustered into 12 distinct subgroups (NAC-I to NAC-XII, **Supplementary Table S2**). The largest clade was subgroup VII with 30 members, whereas the smallest was subgroup XII containing 2 members (**Figure 3** and **Supplementary Table S2**). Noticeably, NAC-X did not include any *Arabidopsis* NAC proteins, which contained 3 poplar members, suggesting that the subfamily may be acquired after their divergence from their common ancestor.

**FIGURE 4:**
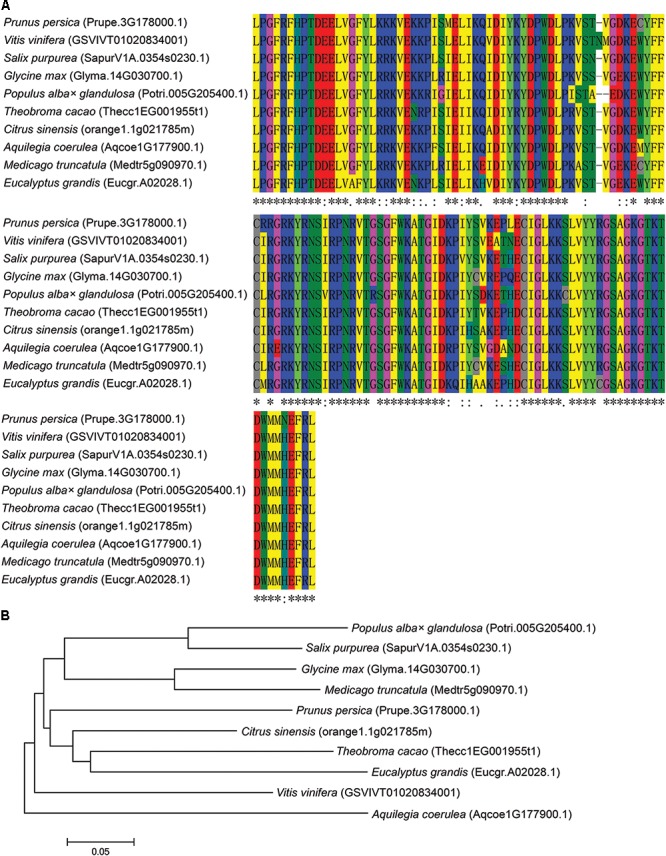
Amino-acid sequence alignment and phylogenetic tree analysis of NACs from P. alba × P. glandulosa and other plant species. **(A*)***, Conserved domain alignment of NACs from different plant species by Clustal W. **(B)**, Phylogenetic tree analysis of NACs from different plant species by MEGA 6.0 with Neighbor-Joining method.

The 1013 bp full-length transcript of *NAC57* gene contains a 5′ untranslated region (16 bp), a 3′ untranslated region (79 bp) and an open reading frame (ORF, 918 bp) encoding 305 amino-acid residues. Multiple amino acids alignment showed that NAC57 protein shared a highly conserved NAC domain (amino acid 24–150) with proteins from other species (**Figure 4A**). BLASTP analysis indicated that the NAC57 (Potri.005G205400.1) protein from *P. alba* × *P. glandulosa* holds 83.3, 74.1, 71.8, 73.4, 73.4, 73.1, 67.2, 69.8, and 66.2% identity with the protein sequences from *Salix purpurea* (SapurV1A.0354s0230.1), *Prunus persica* (Prupe.3G178000.1), *Vitis vinifera* (GSVIVT01020834001), *Glycine max* (Glyma.14G030700.1), *Theobroma cacao* (Thecc1EG001955t1), *Eucalyptus grandis* (Eucgr.A02028.1), *Citrus sinensis* (orange1.1g021785m), *Medicago truncatula* (Medtr5g090970.1), *Aquilegia coerulea* (Aqcoe1G177900.1), respectively. Phylogenetic tree also indicated that the NAC57 from *P. alba* × *P. glandulosa* has relatively high homology with the protein from *S. purpurea*, and relatively distant sequence homology with the protein from *Aquilegia coerulea* (**Figure 4B**).

### Localization of the NAC57 to Cytoplasm and Nucleus

**FIGURE 5:**
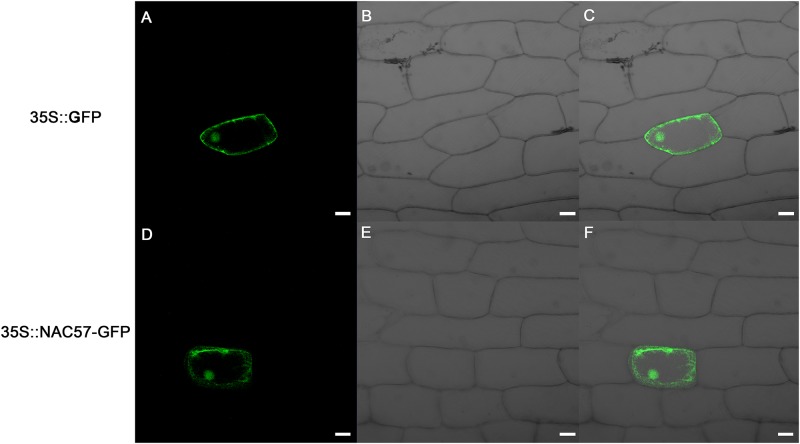
Subcellular localization of NAC57 protein. **(A–C)**, 35S::GFP constructs; **(D–F)**, 35s::NAC57-GFP fusion constructs. **(A,D)**, dark field for green fluorescence; **(B,E)**, bright field; **(C,F)**, overlay of dark field and bright field. Scale bar = 20 μm.

As shown in **Figure 5**, the fluorescence signal of 35S::NAC57-GFP fusion was detected in the cytoplasm and nucleus, similar to control vector 35S::GFP. The results indicated that the subcellular localization of NAC57 protein was not solely nucleus-targeted.

### Salt Tolerance Analysis of Transgenic *Arabidopsis*

We successfully obtained 13 transgenic *Arabidopsis* lines overexpressing *NAC57* gene (TLs) by floral dip method. Three randomly selected T2 TLs and wild type plants (WT) were treated with 0, 100 and 150 mM NaCl for salt tolerance analysis.

**FIGURE 6:**
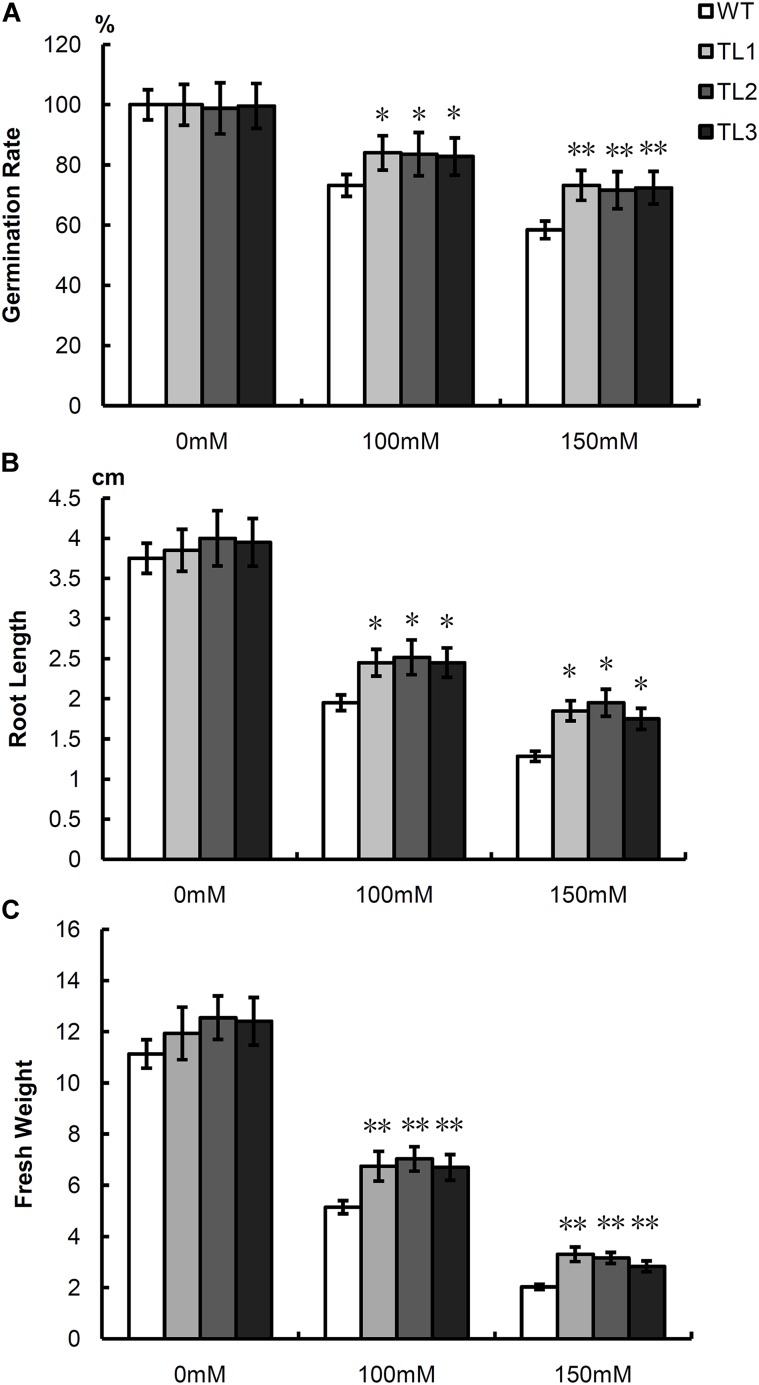
Comparisons of germination rate **(A)**, root length **(B)** and fresh weight **(C)** between TLs and WT under salt stress and normal conditions. WT, wild type; TL1-3, transgenic *Arabidopsis* lines. Mean values and deviations were calculated from three independent biological experiments. ^∗^Indicates significant, ^∗∗^Indicates high significant.

Morphological measurement showed that under normal condition, there was no significant difference between TLs and WT. While under 100 mM NaCl condition, the germination rate, root length and fresh weight of TLs were 1.14, 1.27, and 1.33 times higher than those of WT, respectively. Under 150 mM NaCl condition, the value were 1.24, 1.44, and 1.53, respectively (**Figure 6**).

**FIGURE 7:**
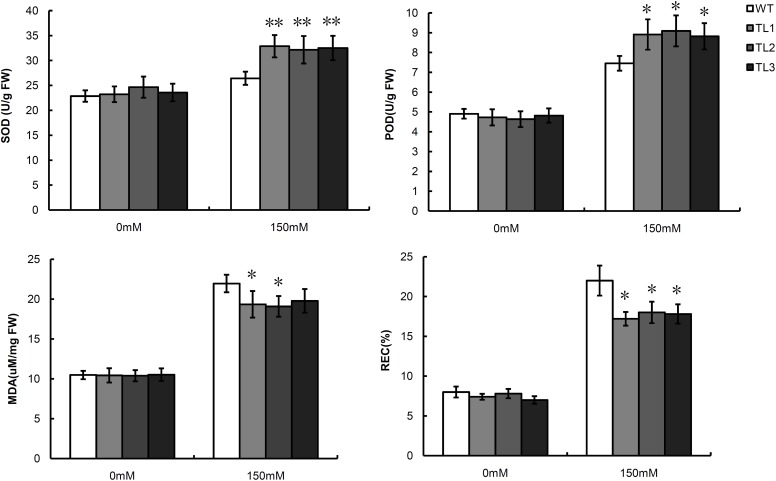
Comparisons of POD activity, SOD activity, MDA content and REC between TLs and WT under salt stress and normal conditions. WT, wild type; TL1-3, transgenic *Arabidopsis* lines. Mean values and deviations were calculated from three independent biological experiments. ^∗^Indicates significant, ^∗∗^Indicates high significant.

Physiological analysis indicated that under normal condition there was no significant difference between TLs and WT. While under 150 mM NaCl condition, POD and SOD activity of the TLs were 1.23 and 1.20 times higher than those of the WT. In contrast to POD and SOD activity, MDA content and REC in the TLs were 1.13 and 1.25 times lower than those in the WT (**Figure 7**).

**FIGURE 8:**
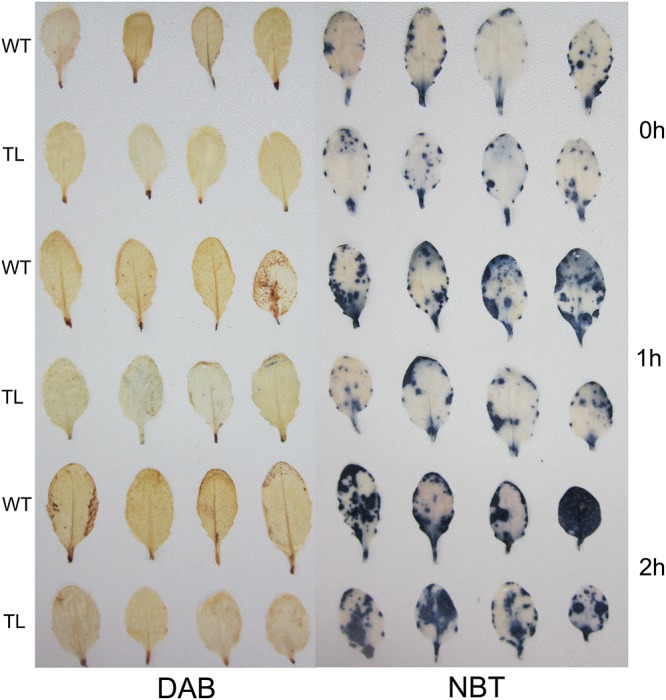
DAB and NBT histochemical staining of *Arabidopsis* plants under salt stress conditions for 0, 1, and 2 h. WT, wild type; TL, transgenic *Arabidopsis* line.

Biochemical staining showed that DAB and NBT coloration in the WT was much darker than those in the TLs (**Figure 8**), indicating ROS accumulation in the WT was more than that in the TLs.

## Discussion

In the present study, we identified a total of 276 up-regulated DEGs and 159 down-regulated DEGs that were shared in *P. alba* × *P. glandulosa* and *P. simonii* × *P. nigra* under salt stress. Among 170 NAC family members, we screened 54 up-regulated and 22 down-regulated NAC genes in *P. alba* × *P. glandulosa* under salt stress, and 61 NAC genes were shared in the two species. In which, *NAC57* gene was a salt-inducible and tissue-specific NAC gene, and salt test of transgenic *Arabidopsis* overexpressing *NAC57* gene indicated the gene plays a significant role in salt tolerance improvement. Comprehensive analyses of *NAC57* gene in the study approve its critical role in salt stress responses and provide fundamental basis for genetically engineering of woody trees.

Complete genome sequences of various model plants, such as *A. thaliana*, *Populus trichocarpa*, *Medicago truncatula*, and many other plant species, provide valuable resources to produce high-quality automatic annotations of plant genes (Foissac et al., 2008). Technical developments in detecting gene expression also facilitate understanding of central roles of genes in abiotic stress responses and plant development. In particular, genome-wide analysis of particular genes or a group of responsive genes, based on high throughput sequencing and computational programs, has become an increasingly popular and comprehensive tool in the field of biological research (Metzker, 2005). Taken woody plants as an example, gene expression dynamics in salt-stressed and unstressed callus from *Populus euphratica* were examined, and a core set of stress-related transcripts were identified by *de novo* assembly of sequenced transcriptome from Solexa data (Qiu et al., 2011). Among 175 ERF genes, as many as 40, 32, 30, 30 genes were identified to be up-regulated in *P. simonii* × *P. nigra* under NaCl, KCl, CdCl2, PEG stress by RNA-Seq, respectively (Yao et al., 2017b). Huang et al. reported a total of 44 NAC genes were strongly up-regulated in *Chrysanthemum lavandulifolium* under salt stress through transcriptome-wide survey (Huang et al., 2012). In the present study, a total of 435 DEGs were identified in *P. alba* × *P. glandulosa* and *P. simonii* × *P. nigra* under salt stress by mRNA abundances profile. As well, 76 salt-inducible NAC genes were screened from RNA-Seq data of *P. alba* × *P. glandulosa*.

Exposed to a variety of abiotic stress conditions, such as salinity, drought, flooding, heat, and cold, plants have evolved a series of physiological and metabolic strategies to respond and adapt (Golldack et al., 2014). One important defense process is called as programmed cell death (PCD), which is initiated to isolate damaged tissues to ensure plant survival (Petrov et al., 2015). PCD process includes an increase in toxic by-product accumulation of cellular metabolisms, namely ROS, which is utilized as signals to modulate plant stress responses (Gechev et al., 2006). ROS exists in various forms, including hydrogen peroxide (H_2_O_2_), superoxide anions (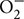), hydroxyl radical (OH•) and singlet oxygen (^1^O_2_), which are mainly controlled by enzymatic antioxidant defense systems (Baxter et al., 2014). SOD and POD are two types of key antioxidant enzymes in ROS-scavenging (Peng et al., 2014; You and Chan, 2015). In particular, as a decomposition product of polyunsaturated fatty acids, increased MDA content indicates more ROS accumulation in plant cells (Yao et al., 2016a). Histochemical staining also can be used to detect ROS accumulation with DAB and NBT as chromogenic substrates (Yao et al., 2016a). In addition, higher REC in plant indicates more damage in plant membrane under stress conditions (Yao et al., 2016a). Besides our previous study (Yao et al., 2016a), there are other reports which prove that TFs confer stress tolerance by deterring ROS accumulation in transgenic plants. For example, the activation of a salt-induced *EIN3/EIL1* gene, precluded excess ROS accumulation and improved salt tolerance in transgenic *Arabidopsis* (Peng et al., 2014). In this study, transgenic *Arabidopsis* overexpressing *NAC57* gene not only showed morphological advantages in germination rate, root length and fresh weight, but also displayed higher POD and SOD activities than WT when challenged with salt stress. Moreover, MDA content determination and histochemical staining indicated there was more ROS accumulation in the WT than that in the TLs. The REC results also showed WT had more damage than TLs. All the results demonstrated that the *NAC57* gene plays a positive role in salt tolerance by enhancing ROS-scavenging capability of transgenic *Arabidopsis*. A phylogenetic tree was constructed with deduced protein sequences of 170 NAC TFs from *Populus trichocarpa* and 124 from *Arabidopsis*. As shown in **Figure 3**, *NAC57* gene (Potri.005G205400.1) belongs to I subgroup of NAC family and has a relatively close homology with a ROS-responsive NAC TF, *ANAC042* gene (AT2G43000.1) from *Arabidopsis*, which regulates longevity in *Arabidopsis* (Wu et al., 2012). *ANAC042* gene was also identified to be involved in the regulation of camalexin biosynthesis in *Arabidopsis* (Saga et al., 2012). This subgroup also contains another NAC TF, *ANAC009* gene (AT1G26870.1), which was reported to regulate periclinal cell division in stem cells (Willemsen et al., 2008). The phylogenetic analysis also indicated the *NAC57* gene was associated with ROS-scavenging and plant developmental processes indirectly.

## Conclusion

In conclusion, we identified a total of 435 DEGs, including 276 up-regulated genes and 159 down-regulated genes in *P. alba* × *P. glandulosa* and *P. simonii* × *P. nigra*, which are associated with stress responses and plant development. As many as 76 NAC genes were identified to be salt responsive in *P. alba* × *P. glandulosa*, including 54 up-regulated and 22 down-regulated genes. Among them, *NAC57* gene was tissue-specific and showed temporal and spatial responses to salt stress. Importantly, overexpression of *NAC57* gene improved salt tolerance in transgenic *Arabidopsis* by deterring ROS accumulation. These results demonstrated that the *NAC57* gene plays an important role in salt stress response.

## Author Contributions

TJ and BZ designed the research. WY conducted the data analysis and wrote the manuscript. KZ, ZC, and XL conducted the experiments. All authors approved the manuscript.

## Conflict of Interest Statement

The authors declare that the research was conducted in the absence of any commercial or financial relationships that could be construed as a potential conflict of interest.

## Footnotes

1 https://www.genewiz.com/

2 http://sys.bio.mtu.edu/index.php

3 http://planttfdb.cbi.pku.edu.cn/

4 https://phytozome.jgi.doe.gov/pz/portal.html

## Supplementary Material

TABLE S1 List of the related primer sequences.

TABLE S2 The information of 170 poplar NAC genes.

DATA SHEET S1 The FPKM of six biological groups of poplar leaves.

DATA SHEET S2 The information of 2198 DEGs and 435 salt-responsive DEGs including 276 up-regulated genes and 159 down-regulated genes.

## Abbreviations

DAB: diaminobenzidine
DEGs: differentially expressed genes
MDA: malondialdehyde
NBT: Nitro blue tetrazolium chloride
POD: peroxidase
REC: relative electrical conductivity
ROS: reactive oxygen species
SOD: superoxide dismutase
TF: transcription factor
TL: transgenic line
WT: wild type.
