# Supplementary material for: Transcriptome Analysis of Poplar Under Salt Stress and Over-Expression of Transcription Factor NAC57 Gene Confers Salt Tolerance in Transgenic Arabidopsis
Source: Front Plant Sci. 2018 Sep 4;9:1121. doi: 10.3389/fpls.2018.01121 (PMC6131821; doi:10.3389/fpls.2018.01121)
Supplement: TABLE S1 — List of the related primer sequences. [file Table_1.DOCX]

Supplemental Table 1 The primer sequence

| Primer Name | Forward primers | Reverse primers |
| --- | --- | --- |
| ACT | ACCCTCCAATCCAGACACTG | TTGCTGACCGTATGAGCAAG |
| EF | AAGCCATGGGATGATGAGAC | ACTGGAGCCAATTTTGATGC |
| PtrNAC57-1 | TCTACAAATACGATCCATGG | GGCCTTCCAAAATCCAGATC |
| PtrNAC57-2 | GCGTCTAGAATGGAGGCCATGACAAAGATGAG | GCGACTAGTCCATCTACATTCGTCAGGAACTTG |
| PtrNAC57-3 | GCGTCTAGAATAGGAGAGAGAGAGAGATGGAGG | GCGGAGCTCAAGCCATCGTATGCTTTTAGAGTTTC |

Note: The underlined sequences stand for restriction enzyme sites.
