# Supplementary material for: Transcriptome Analysis of Poplar Under Salt Stress and Over-Expression of Transcription Factor NAC57 Gene Confers Salt Tolerance in Transgenic Arabidopsis
Source: Front Plant Sci. 2018 Sep 4;9:1121. doi: 10.3389/fpls.2018.01121 (PMC6131821; doi:10.3389/fpls.2018.01121)
Supplement: TABLE S2 — The information of 170 poplar NAC genes. [file Table_2.docx]

TABLE **|** S2 The information of 170 poplar NAC genes

| Gene symbol | Gene ID | *Arabidopsis* ortholog | Biological Processes |
| --- | --- | --- | --- |
| *NAC1* | Potri.001G061200.1 | [AT5G13180.1](http://planttfdb.cbi.pku.edu.cn/tf.php?sp=Ath&did=AT5G13180.1) | response to salt stress and abscisic acid;xylem development; leaf senescence |
| *NAC2* | Potri.001G080900.1 | [AT2G43000.1](http://planttfdb.cbi.pku.edu.cn/tf.php?sp=Ath&did=AT2G43000.1) | regulation of transcription, DNA-templated |
| *NAC3* | Potri.001G120000.1 | [AT1G12260.1](http://planttfdb.cbi.pku.edu.cn/tf.php?sp=Ath&did=AT1G12260.1) | regulation of transcription, DNA-templated;  xylem vessel member cell differentiation; positive regulation of secondary cell wall biogenesis |
| *NAC4* | Potri.001G144400.1 | [AT4G17980.1](http://planttfdb.cbi.pku.edu.cn/tf.php?sp=Ath&did=AT4G17980.1) | regulation of transcription, DNA-templated; cell proliferation; cellular response to auxin stimulus |
| *NAC5* | Potri.001G206900.1 | [AT5G64530.1](http://planttfdb.cbi.pku.edu.cn/tf.php?sp=Ath&did=AT5G64530.1) | regulation of transcription, DNA-templated |
| *NAC6* | Potri.001G218800.1 | [AT3G44350.2](http://planttfdb.cbi.pku.edu.cn/tf.php?sp=Ath&did=AT3G44350.2) | regulation of transcription, DNA-templated; response to chitin |
| *NAC7* | Potri.001G220500.1 | [AT1G77450.1](http://planttfdb.cbi.pku.edu.cn/tf.php?sp=Ath&did=AT1G77450.1) | regulation of transcription, DNA-templated |
| *NAC8* | Potri.001G256600.1 | [AT3G04070.2](http://planttfdb.cbi.pku.edu.cn/tf.php?sp=Ath&did=AT3G04070.2) | regulation of transcription, DNA-templated |
| *NAC9* | Potri.001G325100.1 | [AT5G13180.1](http://planttfdb.cbi.pku.edu.cn/tf.php?sp=Ath&did=AT5G13180.1) | regulation of transcription, DNA-templated |
| *NAC10* | Potri.001G343800.1 | [AT3G01600.1](http://planttfdb.cbi.pku.edu.cn/tf.php?sp=Ath&did=AT3G01600.1) | regulation of transcription, DNA-templated |
| *NAC11* | Potri.001G396300.1 | [AT5G53950.1](http://planttfdb.cbi.pku.edu.cn/tf.php?sp=Ath&did=AT5G53950.1) | regulation of transcription, DNA-templated ;primary shoot apical meristem specification; formation of organ boundary; secondary shoot formation; leaf development; regulation of timing of organ formation |
| *NAC12* | Potri.001G396400.1 | [AT5G53950.1](http://planttfdb.cbi.pku.edu.cn/tf.php?sp=Ath&did=AT5G53950.1) | regulation of transcription, DNA-templated; primary shoot apical meristem specification; formation of organ boundary; secondary shoot formation; leaf development; regulation of timing of organ formation |
| *NAC13* | Potri.001G404100.1 | [AT4G27410.2](http://planttfdb.cbi.pku.edu.cn/tf.php?sp=Ath&did=AT4G27410.2) | regulation of transcription, DNA-templated |
| *NAC14* | Potri.001G404400.1 | [AT3G15510.1](http://planttfdb.cbi.pku.edu.cn/tf.php?sp=Ath&did=AT3G15510.1) | response to jasmonic acid; regulation of embryonic development; seed morphogenesis; integument development |
| *NAC15* | Potri.001G448400.1 | [AT1G32770.1](http://planttfdb.cbi.pku.edu.cn/tf.php?sp=Ath&did=AT1G32770.1) | regulation of transcription, DNA-templated |
| *NAC16* | Potri.001G452700.1 | [AT1G33060.1](http://planttfdb.cbi.pku.edu.cn/tf.php?sp=Ath&did=AT1G33060.1) | regulation of transcription, DNA-templated |
| *NAC17* | Potri.002G005800.1 | [AT1G76420.1](http://planttfdb.cbi.pku.edu.cn/tf.php?sp=Ath&did=AT1G76420.1) | regulation of transcription, DNA-templated;meristem initiation; organ boundary specification between lateral organs and the meristem |
| *NAC18* | Potri.002G037100.1 | [AT4G28530.1](http://planttfdb.cbi.pku.edu.cn/tf.php?sp=Ath&did=AT4G28530.1) | regulation of transcription, DNA-templated |
| *NAC19* | Potri.002G057200.1 | [AT2G43000.1](http://planttfdb.cbi.pku.edu.cn/tf.php?sp=Ath&did=AT2G43000.1) | trehalose biosynthetic process; regulation of transcription, DNA-templated; proline biosynthetic process; anthocyanin-containing compound biosynthetic process; camalexin biosynthetic process; hyperosmotic salinity response; negative regulation of leaf senescence |
| *NAC20* | Potri.002G061300.1 | [AT1G34190.1](http://planttfdb.cbi.pku.edu.cn/tf.php?sp=Ath&did=AT1G34190.1) | regulation of transcription, DNA-templated; cellular response to hydrogen peroxide |
| *NAC21* | Potri.002G081000.1 | [AT1G01720.1](http://planttfdb.cbi.pku.edu.cn/tf.php?sp=Ath&did=AT1G01720.1) | regulation of transcription, DNA-templated;response to wounding; negative regulation of abscisic acid-activated signaling pathway |
| *NAC22* | Potri.002G154000.1 | [AT1G33060.2](http://planttfdb.cbi.pku.edu.cn/tf.php?sp=Ath&did=AT1G33060.2) | regulation of transcription, DNA-templated |
| *NAC23* | Potri.002G154100.1 | [AT1G33060.2](http://planttfdb.cbi.pku.edu.cn/tf.php?sp=Ath&did=AT1G33060.2) | regulation of transcription, DNA-templated |
| *NAC24* | Potri.002G154200.1 | [AT4G35580.1](http://planttfdb.cbi.pku.edu.cn/tf.php?sp=Ath&did=AT4G35580.1) | regulation of transcription, DNA-templated |
| *NAC25* | Potri.002G178700.1 | [AT2G46770.1](http://planttfdb.cbi.pku.edu.cn/tf.php?sp=Ath&did=AT2G46770.1) | regulation of transcription, DNA-templated |
| *NAC26* | Potri.002G181900.1 | [AT4G01550.1](http://planttfdb.cbi.pku.edu.cn/tf.php?sp=Ath&did=AT4G01550.1) | regulation of transcription, DNA-templated |
| *NAC27* | Potri.002G182000.1 | [AT4G35580.1](http://planttfdb.cbi.pku.edu.cn/tf.php?sp=Ath&did=AT4G35580.1) | regulation of transcription, DNA-templated |
| *NAC28* | Potri.002G182300.1 | [AT4G35580.1](http://planttfdb.cbi.pku.edu.cn/tf.php?sp=Ath&did=AT4G35580.1) | regulation of transcription, DNA-templated |
| *NAC29* | Potri.002G182400.1 | [AT4G35580.3](http://planttfdb.cbi.pku.edu.cn/tf.php?sp=Ath&did=AT4G35580.3) | regulation of transcription, DNA-templated |
| *NAC30* | Potri.003G022800.1 | [AT5G64530.1](http://planttfdb.cbi.pku.edu.cn/tf.php?sp=Ath&did=AT5G64530.1) | regulation of transcription, DNA-templated; xylem development; regulation of programmed cell death; shoot system development |
| *NAC31* | Potri.003G046700.1 | [AT2G02450.1](http://planttfdb.cbi.pku.edu.cn/tf.php?sp=Ath&did=AT2G02450.1) | regulation of transcription, DNA-templated |
| *NAC32* | Potri.003G089800.1 | [AT4G17980.1](http://planttfdb.cbi.pku.edu.cn/tf.php?sp=Ath&did=AT4G17980.1) | regulation of transcription, DNA-templated;cell proliferation; cellular response to auxin stimulus |
| *NAC33* | Potri.003G103500.1 | [AT1G69490.1](http://planttfdb.cbi.pku.edu.cn/tf.php?sp=Ath&did=AT1G69490.1) | regulation of transcription, DNA-templated |
| *NAC34* | Potri.003G113000.1 | [AT1G12260.1](http://planttfdb.cbi.pku.edu.cn/tf.php?sp=Ath&did=AT1G12260.1) | regulation of transcription, DNA-templated |
| *NAC35* | Potri.003G149400.1 | [AT3G04070.2](http://planttfdb.cbi.pku.edu.cn/tf.php?sp=Ath&did=AT3G04070.2) | regulation of transcription, DNA-templated |
| *NAC36* | Potri.003G149700.1 | [AT2G43000.1](http://planttfdb.cbi.pku.edu.cn/tf.php?sp=Ath&did=AT2G43000.1) | regulation of transcription, DNA-templated |
| *NAC37* | Potri.003G166500.1 | [AT5G13180.1](http://planttfdb.cbi.pku.edu.cn/tf.php?sp=Ath&did=AT5G13180.1) | response to salt stress; response to abscisic acid;xylem development; leaf senescence |
| *NAC38* | Potri.004G038000.1 | [AT1G61110.1](http://planttfdb.cbi.pku.edu.cn/tf.php?sp=Ath&did=AT1G61110.1) | regulation of transcription, DNA-templated |
| *NAC39* | Potri.004G049300.1 | [AT4G28500.1](http://planttfdb.cbi.pku.edu.cn/tf.php?sp=Ath&did=AT4G28500.1) | regulation of transcription, DNA-templated |
| *NAC40* | Potri.004G049500.1 | [AT1G61110.1](http://planttfdb.cbi.pku.edu.cn/tf.php?sp=Ath&did=AT1G61110.1) | regulation of transcription, DNA-templated |
| *NAC41* | Potri.004G081000.1 | [AT1G65910.1](http://planttfdb.cbi.pku.edu.cn/tf.php?sp=Ath&did=AT1G65910.1) | regulation of transcription, DNA-templated |
| *NAC42* | Potri.004G107200.1 | [AT5G62380.1](http://planttfdb.cbi.pku.edu.cn/tf.php?sp=Ath&did=AT5G62380.1) | regulation of transcription, DNA-templated |
| *NAC43* | Potri.004G107400.1 | [AT5G62380.1](http://planttfdb.cbi.pku.edu.cn/tf.php?sp=Ath&did=AT5G62380.1) | regulation of transcription, DNA-templated |
| *NAC44* | Potri.004G119400.1 | [AT1G69490.1](http://planttfdb.cbi.pku.edu.cn/tf.php?sp=Ath&did=AT1G69490.1) | regulation of transcription, DNA-templated |
| *NAC45* | Potri.004G181900.1 | [AT2G17040.1](http://planttfdb.cbi.pku.edu.cn/tf.php?sp=Ath&did=AT2G17040.1) | regulation of transcription, DNA-templated; leaf morphogenesis; response to chitin; negative regulation of cell size; inflorescence morphogenesis |
| *NAC46* | Potri.004G230800.1 | [AT2G02450.1](http://planttfdb.cbi.pku.edu.cn/tf.php?sp=Ath&did=AT2G02450.1) | regulation of transcription, DNA-templated |
| *NAC47* | Potri.005G058900.1 | [AT5G64060.1](http://planttfdb.cbi.pku.edu.cn/tf.php?sp=Ath&did=AT5G64060.1) | regulation of transcription, DNA-templated |
| *NAC48* | Potri.005G064100.1 | [AT5G64530.1](http://planttfdb.cbi.pku.edu.cn/tf.php?sp=Ath&did=AT5G64530.1) | regulation of transcription, DNA-templated |
| *NAC49* | Potri.005G069500.1 | [AT1G01720.1](http://planttfdb.cbi.pku.edu.cn/tf.php?sp=Ath&did=AT1G01720.1) | regulation of transcription, DNA-templated |
| *NAC50* | Potri.005G082700.1 | [AT5G62380.1](http://planttfdb.cbi.pku.edu.cn/tf.php?sp=Ath&did=AT5G62380.1) | regulation of transcription, DNA-templated |
| *NAC51* | Potri.005G098000.1 | [AT3G12910.1](http://planttfdb.cbi.pku.edu.cn/tf.php?sp=Ath&did=AT3G12910.1) | regulation of transcription, DNA-templated |
| *NAC52* | Potri.005G098200.1 | [AT1G56010.2](http://planttfdb.cbi.pku.edu.cn/tf.php?sp=Ath&did=AT1G56010.2) | regulation of transcription, DNA-templated |
| *NAC53* | Potri.005G103200.1 | [AT2G17040.1](http://planttfdb.cbi.pku.edu.cn/tf.php?sp=Ath&did=AT2G17040.1) | regulation of transcription, DNA-templated |
| *NAC54* | Potri.005G116800.1 | [AT2G18060.1](http://planttfdb.cbi.pku.edu.cn/tf.php?sp=Ath&did=AT2G18060.1) | regulation of transcription, DNA-templated |
| *NAC55* | Potri.005G180200.1 | [AT1G01720.1](http://planttfdb.cbi.pku.edu.cn/tf.php?sp=Ath&did=AT1G01720.1) | regulation of transcription, DNA-templated |
| *NAC56* | Potri.005G200100.1 | [AT1G34190.1](http://planttfdb.cbi.pku.edu.cn/tf.php?sp=Ath&did=AT1G34190.1) | regulation of transcription, DNA-templated |
| *NAC57* | Potri.005G205400.1 | [AT2G43000.1](http://planttfdb.cbi.pku.edu.cn/tf.php?sp=Ath&did=AT2G43000.1) | regulation of transcription, DNA-templated |
| *NAC58* | Potri.005G225800.1 | [AT4G28530.1](http://planttfdb.cbi.pku.edu.cn/tf.php?sp=Ath&did=AT4G28530.1) | regulation of transcription, DNA-templated |
| *NAC59* | Potri.005G255900.1 | [AT1G76420.1](http://planttfdb.cbi.pku.edu.cn/tf.php?sp=Ath&did=AT1G76420.1) | regulation of transcription, DNA-templated |
| *NAC60* | Potri.006G028300.1 | [AT3G10480.2](http://planttfdb.cbi.pku.edu.cn/tf.php?sp=Ath&did=AT3G10480.2) | regulation of transcription, DNA-templated |
| *NAC61* | Potri.006G028700.1 | [AT2G24430.2](http://planttfdb.cbi.pku.edu.cn/tf.php?sp=Ath&did=AT2G24430.2) | regulation of transcription, DNA-templated |
| *NAC62* | Potri.006G028900.1 | [AT3G10480.2](http://planttfdb.cbi.pku.edu.cn/tf.php?sp=Ath&did=AT3G10480.2) | regulation of transcription, DNA-templated |
| *NAC63* | Potri.006G029200.1 | [AT3G10480.2](http://planttfdb.cbi.pku.edu.cn/tf.php?sp=Ath&did=AT3G10480.2) | regulation of transcription, DNA-templated |
| *NAC64* | Potri.006G029300.1 | [AT5G24590.2](http://planttfdb.cbi.pku.edu.cn/tf.php?sp=Ath&did=AT5G24590.2) | regulation of transcription, DNA-templated |
| *NAC65* | Potri.006G029600.1 | [AT3G10480.2](http://planttfdb.cbi.pku.edu.cn/tf.php?sp=Ath&did=AT3G10480.2) | regulation of transcription, DNA-templated |
| *NAC66* | Potri.006G030600.1 | [AT3G10480.2](http://planttfdb.cbi.pku.edu.cn/tf.php?sp=Ath&did=AT3G10480.2) | regulation of transcription, DNA-templated |
| *NAC67* | Potri.006G030800.1 | [AT3G10480.2](http://planttfdb.cbi.pku.edu.cn/tf.php?sp=Ath&did=AT3G10480.2) | regulation of transcription, DNA-templated |
| *NAC68* | Potri.006G051400.1 | [AT3G04070.2](http://planttfdb.cbi.pku.edu.cn/tf.php?sp=Ath&did=AT3G04070.2) | regulation of transcription, DNA-templated |
| *NAC69* | Potri.006G129400.1 | [AT1G61110.1](http://planttfdb.cbi.pku.edu.cn/tf.php?sp=Ath&did=AT1G61110.1) | regulation of transcription, DNA-templated |
| *NAC70* | Potri.006G152700.1 | [AT4G29230.1](http://planttfdb.cbi.pku.edu.cn/tf.php?sp=Ath&did=AT4G29230.1) | regulation of transcription, DNA-templated |
| *NAC71* | Potri.006G179800.1 | [AT3G44350.1](http://planttfdb.cbi.pku.edu.cn/tf.php?sp=Ath&did=AT3G44350.1) | regulation of transcription, DNA-templated |
| *NAC72* | Potri.006G209200.1 | [AT5G22380.1](http://planttfdb.cbi.pku.edu.cn/tf.php?sp=Ath&did=AT5G22380.1) | regulation of transcription, DNA-templated |
| *NAC73* | Potri.006G231300.1 | [AT5G62380.1](http://planttfdb.cbi.pku.edu.cn/tf.php?sp=Ath&did=AT5G62380.1) | regulation of transcription, DNA-templated |
| *NAC74* | Potri.006G277000.1 | [AT2G24430.2](http://planttfdb.cbi.pku.edu.cn/tf.php?sp=Ath&did=AT2G24430.2) | regulation of transcription, DNA-templated |
| *NAC75* | Potri.007G014400.1 | [AT2G18060.1](http://planttfdb.cbi.pku.edu.cn/tf.php?sp=Ath&did=AT2G18060.1) | regulation of transcription, DNA-templated；response to chitin；xylem vessel member cell differentiation；positive regulation of secondary cell wall biogenesis；callus formation |
| *NAC76* | Potri.007G065400.1 | [AT1G56010.2](http://planttfdb.cbi.pku.edu.cn/tf.php?sp=Ath&did=AT1G56010.2) | regulation of transcription, DNA-templated |
| *NAC77* | Potri.007G066300.1 | [AT3G12910.1](http://planttfdb.cbi.pku.edu.cn/tf.php?sp=Ath&did=AT3G12910.1) | regulation of transcription, DNA-templated |
| *NAC78* | Potri.007G099400.1 | [AT1G01720.1](http://planttfdb.cbi.pku.edu.cn/tf.php?sp=Ath&did=AT1G01720.1) | regulation of transcription, DNA-templated |
| *NAC79* | Potri.007G105000.1 | [AT5G64530.1](http://planttfdb.cbi.pku.edu.cn/tf.php?sp=Ath&did=AT5G64530.1) | regulation of transcription, DNA-templated |
| *NAC80* | Potri.007G109100.1 | [AT5G09330.4](http://planttfdb.cbi.pku.edu.cn/tf.php?sp=Ath&did=AT5G09330.4) | regulation of transcription, DNA-templated |
| *NAC81* | Potri.007G127700.1 | [AT5G13180.1](http://planttfdb.cbi.pku.edu.cn/tf.php?sp=Ath&did=AT5G13180.1) | regulation of transcription, DNA-templated |
| *NAC82* | Potri.007G135300.1 | [AT4G28500.1](http://planttfdb.cbi.pku.edu.cn/tf.php?sp=Ath&did=AT4G28500.1) | regulation of transcription, DNA-templated |
| *NAC83* | Potri.008G031800.1 | [AT5G04410.1](http://planttfdb.cbi.pku.edu.cn/tf.php?sp=Ath&did=AT5G04410.1) | response to high light intensity; regulation of flavonoid biosynthetic process; positive regulation of transcription, DNA-templated |
| *NAC84* | Potri.008G080000.1 | [AT1G79580.3](http://planttfdb.cbi.pku.edu.cn/tf.php?sp=Ath&did=AT1G79580.3) | regulation of transcription, DNA-templated |
| *NAC85* | Potri.008G081500.1 | [AT1G54330.1](http://planttfdb.cbi.pku.edu.cn/tf.php?sp=Ath&did=AT1G54330.1) | regulation of transcription, DNA-templated |
| *NAC86* | Potri.008G089000.1 | [AT1G69490.1](http://planttfdb.cbi.pku.edu.cn/tf.php?sp=Ath&did=AT1G69490.1) | regulation of transcription, DNA-templated; multidimensional cell growth; fruit ripening；flower development; leaf senescence |
| *NAC87* | Potri.008G116600.1 | [AT1G25580.1](http://planttfdb.cbi.pku.edu.cn/tf.php?sp=Ath&did=AT1G25580.1) | DNA damage checkpoint;regulation of transcription, DNA-templated; response to gamma radiation; regulation of meiotic nuclear division |
| *NAC88* | Potri.009G019200.1 | [AT3G44350.2](http://planttfdb.cbi.pku.edu.cn/tf.php?sp=Ath&did=AT3G44350.2) | regulation of transcription, DNA-templated |
| *NAC89* | Potri.009G052200.1 | [AT4G27410.2](http://planttfdb.cbi.pku.edu.cn/tf.php?sp=Ath&did=AT4G27410.2) | regulation of transcription, DNA-templated |
| *NAC90* | Potri.009G052300.1 | [AT1G01720.1](http://planttfdb.cbi.pku.edu.cn/tf.php?sp=Ath&did=AT1G01720.1) | regulation of transcription, DNA-templated |
| *NAC91* | Potri.009G072100.1 | [AT4G17980.1](http://planttfdb.cbi.pku.edu.cn/tf.php?sp=Ath&did=AT4G17980.1) | regulation of transcription, DNA-templated |
| *NAC92* | Potri.009G141600.1 | [AT2G17040.1](http://planttfdb.cbi.pku.edu.cn/tf.php?sp=Ath&did=AT2G17040.1) | regulation of transcription, DNA-templated |
| *NAC93* | Potri.009G161300.1 | [AT2G27300.1](http://planttfdb.cbi.pku.edu.cn/tf.php?sp=Ath&did=AT2G27300.1) | regulation of transcription, DNA-templated ;seed germination  ; negative regulation of gibberellic acid mediated signaling pathway ;membrane protein proteolysis ;photoperiodism, flowering ;cellular response to salt stress |
| *NAC94* | Potri.010G129700.1 | [AT1G25580.1](http://planttfdb.cbi.pku.edu.cn/tf.php?sp=Ath&did=AT1G25580.1) | regulation of transcription, DNA-templated |
| *NAC95* | Potri.010G166200.1 | [AT1G69490.1](http://planttfdb.cbi.pku.edu.cn/tf.php?sp=Ath&did=AT1G69490.1) | regulation of transcription, DNA-templated |
| *NAC96* | Potri.010G174600.1 | [AT1G54330.1](http://planttfdb.cbi.pku.edu.cn/tf.php?sp=Ath&did=AT1G54330.1) | regulation of transcription, DNA-templated |
| *NAC97* | Potri.010G176600.1 | [AT1G79580.3](http://planttfdb.cbi.pku.edu.cn/tf.php?sp=Ath&did=AT1G79580.3) | regionalization; regulation of transcription, DNA-templated; plant-type secondary cell wall biogenesis; positive regulation of cell fate commitment; root cap development |
| *NAC98* | Potri.010G229700.1 | [AT3G10480.1](http://planttfdb.cbi.pku.edu.cn/tf.php?sp=Ath&did=AT3G10480.1) | regulation of transcription, DNA-templated; pollen development; vegetative to reproductive phase transition of meristem |
| *NAC99* | Potri.010G229900.1 | [AT5G04410.1](http://planttfdb.cbi.pku.edu.cn/tf.php?sp=Ath&did=AT5G04410.1) | regulation of transcription, DNA-templated |
| *NAC100* | Potri.011G046700.1 | [AT1G61110.1](http://planttfdb.cbi.pku.edu.cn/tf.php?sp=Ath&did=AT1G61110.1) | regulation of transcription, DNA-templated |
| *NAC101* | Potri.011G058400.1 | [AT4G28500.1](http://planttfdb.cbi.pku.edu.cn/tf.php?sp=Ath&did=AT4G28500.1) | regulation of transcription, DNA-templated |
| *NAC102* | Potri.011G058600.1 | [AT3G15510.1](http://planttfdb.cbi.pku.edu.cn/tf.php?sp=Ath&did=AT3G15510.1) | regulation of transcription, DNA-templated |
| *NAC103* | Potri.011G115400.1 | [AT5G53950.1](http://planttfdb.cbi.pku.edu.cn/tf.php?sp=Ath&did=AT5G53950.1) | regulation of transcription, DNA-templated |
| *NAC104* | Potri.011G121300.1 | [AT5G17260.1](http://planttfdb.cbi.pku.edu.cn/tf.php?sp=Ath&did=AT5G17260.1) | regulation of transcription, DNA-templated |
| *NAC105* | Potri.011G123300.1 | [AT4G27410.2](http://planttfdb.cbi.pku.edu.cn/tf.php?sp=Ath&did=AT4G27410.2) | response to water deprivation; response to abscisic acid; positive regulation of transcription, DNA-templated |
| *NAC106* | Potri.011G123500.1 | [AT3G15510.1](http://planttfdb.cbi.pku.edu.cn/tf.php?sp=Ath&did=AT3G15510.1) | regulation of transcription, DNA-templated |
| *NAC107* | Potri.011G149300.1 | [AT1G33060.1](http://planttfdb.cbi.pku.edu.cn/tf.php?sp=Ath&did=AT1G33060.1) | regulation of transcription, DNA-templated |
| *NAC108* | Potri.011G153300.1 | [AT2G46770.1](http://planttfdb.cbi.pku.edu.cn/tf.php?sp=Ath&did=AT2G46770.1) | lignin biosynthetic process; plant-type secondary cell wall biogenesis; fruit dehiscence; positive regulation of transcription, DNA-templated |
| *NAC109* | Potri.012G001400.1 | [AT5G61430.1](http://planttfdb.cbi.pku.edu.cn/tf.php?sp=Ath&did=AT5G61430.1) | regulation of transcription, DNA-templated |
| *NAC110* | Potri.012G007500.1 | [AT5G24590.2](http://planttfdb.cbi.pku.edu.cn/tf.php?sp=Ath&did=AT5G24590.2) | positive regulation of defense response to virus by host;response to molecule of bacterial origin;regulation of transcription, DNA-templated;defense response, incompatible interaction;response to chitin;cellular response to cold |
| *NAC111* | Potri.012G023900.1 | [AT5G17260.1](http://planttfdb.cbi.pku.edu.cn/tf.php?sp=Ath&did=AT5G17260.1) | regulation of transcription, DNA-templated |
| *NAC112* | Potri.012G024100.1 | [AT5G17260.1](http://planttfdb.cbi.pku.edu.cn/tf.php?sp=Ath&did=AT5G17260.1) | regulation of transcription, DNA-templated |
| *NAC113* | Potri.012G024200.1 | [AT5G17260.1](http://planttfdb.cbi.pku.edu.cn/tf.php?sp=Ath&did=AT5G17260.1) | regulation of transcription, DNA-templated |
| *NAC114* | Potri.012G038100.1 | [AT3G17730.1](http://planttfdb.cbi.pku.edu.cn/tf.php?sp=Ath&did=AT3G17730.1) | regulation of transcription, DNA-templated |
| *NAC115* | Potri.012G056300.1 | [AT3G18400.1](http://planttfdb.cbi.pku.edu.cn/tf.php?sp=Ath&did=AT3G18400.1) | regulation of transcription, DNA-templated |
| *NAC116* | Potri.012G103500.1 | [AT5G13180.1](http://planttfdb.cbi.pku.edu.cn/tf.php?sp=Ath&did=AT5G13180.1) | regulation of transcription, DNA-templated |
| *NAC117* | Potri.012G126500.1 | [AT1G12260.1](http://planttfdb.cbi.pku.edu.cn/tf.php?sp=Ath&did=AT1G12260.1) | regulation of transcription, DNA-templated |
| *NAC118* | Potri.013G054000.1 | [AT3G04070.1](http://planttfdb.cbi.pku.edu.cn/tf.php?sp=Ath&did=AT3G04070.1) | regulation of transcription, DNA-templated |
| *NAC119* | Potri.013G054200.1 | [AT5G18270.1](http://planttfdb.cbi.pku.edu.cn/tf.php?sp=Ath&did=AT5G18270.1) | regulation of transcription, DNA-templated |
| *NAC120* | Potri.013G079700.1 | [AT3G17730.1](http://planttfdb.cbi.pku.edu.cn/tf.php?sp=Ath&did=AT3G17730.1) | regulation of transcription, DNA-templated |
| *NAC121* | Potri.013G092400.1 | [AT4G10350.1](http://planttfdb.cbi.pku.edu.cn/tf.php?sp=Ath&did=AT4G10350.1) | regulation of transcription, DNA-templated |
| *NAC122* | Potri.013G113100.1 | [AT1G71930.1](http://planttfdb.cbi.pku.edu.cn/tf.php?sp=Ath&did=AT1G71930.1) | response to cytokinin; response to abscisic acid; response to brassinosteroid; xylan metabolic process; positive regulation of transcription, DNA-templated; xylem vessel member cell differentiation; defense response to fungus; cellular response to auxin stimulus; protoxylem development |
| *NAC123* | Potri.014G025700.1 | [AT1G69490.1](http://planttfdb.cbi.pku.edu.cn/tf.php?sp=Ath&did=AT1G69490.1) | regulation of transcription, DNA-templated |
| *NAC124* | Potri.014G041300.1 | [AT1G65910.1](http://planttfdb.cbi.pku.edu.cn/tf.php?sp=Ath&did=AT1G65910.1) | regulation of transcription, DNA-templated |
| *NAC125* | Potri.014G064600.1 | [AT1G34190.1](http://planttfdb.cbi.pku.edu.cn/tf.php?sp=Ath&did=AT1G34190.1) | regulation of transcription, DNA-templated |
| *NAC126* | Potri.014G075900.1 | [AT4G17980.1](http://planttfdb.cbi.pku.edu.cn/tf.php?sp=Ath&did=AT4G17980.1) | regulation of transcription, DNA-templated |
| *NAC127* | Potri.014G076000.1 | [AT4G35580.3](http://planttfdb.cbi.pku.edu.cn/tf.php?sp=Ath&did=AT4G35580.3) | regulation of transcription, DNA-templated |
| *NAC128* | Potri.014G076100.1 | [AT4G17980.1](http://planttfdb.cbi.pku.edu.cn/tf.php?sp=Ath&did=AT4G17980.1) | regulation of transcription, DNA-templated |
| *NAC129* | Potri.014G104800.1 | [AT2G46770.1](http://planttfdb.cbi.pku.edu.cn/tf.php?sp=Ath&did=AT2G46770.1) | lignin biosynthetic process; plant-type secondary cell wall biogenesis; anther dehiscence; fruit dehiscence; positive regulation of transcription, DNA-templated |
| *NAC130* | Potri.014G107600.1 | [AT4G35580.3](http://planttfdb.cbi.pku.edu.cn/tf.php?sp=Ath&did=AT4G35580.3) | regulation of transcription, DNA-templated |
| *NAC131* | Potri.014G107700.1 | [AT4G35580.1](http://planttfdb.cbi.pku.edu.cn/tf.php?sp=Ath&did=AT4G35580.1) | regulation of transcription, DNA-templated |
| *NAC132* | Potri.014G107900.1 | [AT4G35580.1](http://planttfdb.cbi.pku.edu.cn/tf.php?sp=Ath&did=AT4G35580.1) | regulation of transcription, DNA-templated |
| *NAC133* | Potri.014G108000.1 | [AT4G35580.1](http://planttfdb.cbi.pku.edu.cn/tf.php?sp=Ath&did=AT4G35580.1) | regulation of transcription, DNA-templated |
| *NAC134* | Potri.014G108100.1 | [AT5G17260.1](http://planttfdb.cbi.pku.edu.cn/tf.php?sp=Ath&did=AT5G17260.1) | regulation of transcription, DNA-templated |
| *NAC135* | Potri.014G163600.1 | [AT5G62380.1](http://planttfdb.cbi.pku.edu.cn/tf.php?sp=Ath&did=AT5G62380.1) | regulation of transcription, DNA-templated |
| *NAC136* | Potri.015G002900.1 | [AT1G71930.1](http://planttfdb.cbi.pku.edu.cn/tf.php?sp=Ath&did=AT1G71930.1) | regulation of transcription, DNA-templated |
| *NAC137* | Potri.015G004100.1 | [AT3G49530.1](http://planttfdb.cbi.pku.edu.cn/tf.php?sp=Ath&did=AT3G49530.1) | regulation of transcription, DNA-templated; defense response, incompatible interaction; response to chitin; cellular response to cold |
| *NAC138* | Potri.015G007000.1 | [AT2G33480.2](http://planttfdb.cbi.pku.edu.cn/tf.php?sp=Ath&did=AT2G33480.2) | regulation of transcription, DNA-templated |
| *NAC139* | Potri.015G020000.1 | [AT5G61430.1](http://planttfdb.cbi.pku.edu.cn/tf.php?sp=Ath&did=AT5G61430.1) | regulation of transcription, DNA-templated |
| *NAC140* | Potri.015G030200.1 | [AT3G17730.1](http://planttfdb.cbi.pku.edu.cn/tf.php?sp=Ath&did=AT3G17730.1) | regulation of transcription, DNA-templated |
| *NAC141* | Potri.015G046800.1 | [AT3G18400.1](http://planttfdb.cbi.pku.edu.cn/tf.php?sp=Ath&did=AT3G18400.1) | regulation of transcription, DNA-templated |
| *NAC142* | Potri.015G102100.1 | [AT5G13180.1](http://planttfdb.cbi.pku.edu.cn/tf.php?sp=Ath&did=AT5G13180.1) | regulation of transcription, DNA-templated |
| *NAC143* | Potri.015G127400.1 | [AT1G12260.1](http://planttfdb.cbi.pku.edu.cn/tf.php?sp=Ath&did=AT1G12260.1) | regulation of transcription, DNA-templated |
| *NAC144* | Potri.016G027900.1 | [AT2G24430.2](http://planttfdb.cbi.pku.edu.cn/tf.php?sp=Ath&did=AT2G24430.2) | regulation of transcription, DNA-templated |
| *NAC145* | Potri.016G055500.1 | [AT1G61110.1](http://planttfdb.cbi.pku.edu.cn/tf.php?sp=Ath&did=AT1G61110.1) | regulation of transcription, DNA-templated |
| *NAC146* | Potri.016G076000.1 | [AT5G22380.1](http://planttfdb.cbi.pku.edu.cn/tf.php?sp=Ath&did=AT5G22380.1) | regulation of transcription, DNA-templated |
| *NAC147* | Potri.016G076100.1 | [AT5G22380.1](http://planttfdb.cbi.pku.edu.cn/tf.php?sp=Ath&did=AT5G22380.1) | regulation of transcription, DNA-templated |
| *NAC148* | Potri.016G088300.1 | [AT1G61110.1](http://planttfdb.cbi.pku.edu.cn/tf.php?sp=Ath&did=AT1G61110.1) | regulation of transcription, DNA-templated |
| *NAC149* | Potri.017G016700.1 | [AT4G28500.1](http://planttfdb.cbi.pku.edu.cn/tf.php?sp=Ath&did=AT4G28500.1) | positive regulation of transcription, DNA-templated; regulation of secondary cell wall biogenesis |
| *NAC150* | Potri.017G031000.1 | [AT5G13180.1](http://planttfdb.cbi.pku.edu.cn/tf.php?sp=Ath&did=AT5G13180.1) | regulation of transcription, DNA-templated |
| *NAC151* | Potri.017G031300.1 | [AT5G13180.1](http://planttfdb.cbi.pku.edu.cn/tf.php?sp=Ath&did=AT5G13180.1) | regulation of transcription, DNA-templated |
| *NAC152* | Potri.017G031600.1 | [AT2G33480.1](http://planttfdb.cbi.pku.edu.cn/tf.php?sp=Ath&did=AT2G33480.1) | regulation of transcription, DNA-templated |
| *NAC153* | Potri.017G058100.1 | [AT2G18060.1](http://planttfdb.cbi.pku.edu.cn/tf.php?sp=Ath&did=AT2G18060.1) | regulation of transcription, DNA-templated |
| *NAC154* | Potri.017G063300.1 | [AT5G13180.1](http://planttfdb.cbi.pku.edu.cn/tf.php?sp=Ath&did=AT5G13180.1) | regulation of transcription, DNA-templated |
| *NAC155* | Potri.017G082000.1 | [AT1G26870.1](http://planttfdb.cbi.pku.edu.cn/tf.php?sp=Ath&did=AT1G26870.1) | regulation of transcription, DNA-templated |
| *NAC156* | Potri.017G086200.1 | [AT5G61430.1](http://planttfdb.cbi.pku.edu.cn/tf.php?sp=Ath&did=AT5G61430.1) | regulation of transcription, DNA-templated |
| *NAC157* | Potri.017G104900.1 | [AT1G34190.1](http://planttfdb.cbi.pku.edu.cn/tf.php?sp=Ath&did=AT1G34190.1) | regulation of transcription, DNA-templated |
| *NAC158* | Potri.017G139500.1 | [AT1G65910.1](http://planttfdb.cbi.pku.edu.cn/tf.php?sp=Ath&did=AT1G65910.1) | regulation of transcription, DNA-templated |
| *NAC159* | Potri.018G003800.1 | [AT2G24430.2](http://planttfdb.cbi.pku.edu.cn/tf.php?sp=Ath&did=AT2G24430.2) | regulation of transcription, DNA-templated |
| *NAC160* | Potri.018G049300.1 | [AT1G26870.1](http://planttfdb.cbi.pku.edu.cn/tf.php?sp=Ath&did=AT1G26870.1) | regulation of transcription, DNA-templated |
| *NAC161* | Potri.018G068700.1 | [AT4G29230.1](http://planttfdb.cbi.pku.edu.cn/tf.php?sp=Ath&did=AT4G29230.1) | regulation of transcription, DNA-templated |
| *NAC162* | Potri.018G095000.1 | [AT1G01720.1](http://planttfdb.cbi.pku.edu.cn/tf.php?sp=Ath&did=AT1G01720.1) | regulation of transcription, DNA-templated |
| *NAC163* | Potri.019G031400.1 | [AT3G04070.1](http://planttfdb.cbi.pku.edu.cn/tf.php?sp=Ath&did=AT3G04070.1) | regulation of transcription, DNA-templated |
| *NAC164* | Potri.019G031600.1 | [AT5G18270.1](http://planttfdb.cbi.pku.edu.cn/tf.php?sp=Ath&did=AT5G18270.1) | regulation of transcription, DNA-templated |
| *NAC165* | Potri.019G063000.1 | [AT1G79580.3](http://planttfdb.cbi.pku.edu.cn/tf.php?sp=Ath&did=AT1G79580.3) | regulation of transcription, DNA-templated |
| *NAC166* | Potri.019G066000.1 | [AT4G10350.1](http://planttfdb.cbi.pku.edu.cn/tf.php?sp=Ath&did=AT4G10350.1) | plant-type secondary cell wall biogenesis; positive regulation of transcription, DNA-templated; root cap development |
| *NAC167* | Potri.019G083600.1 | [AT1G71930.1](http://planttfdb.cbi.pku.edu.cn/tf.php?sp=Ath&did=AT1G71930.1) | regulation of transcription, DNA-templated |
| *NAC168* | Potri.019G099800.1 | [AT5G24590.2](http://planttfdb.cbi.pku.edu.cn/tf.php?sp=Ath&did=AT5G24590.2) | regulation of transcription, DNA-templated |
| *NAC169* | Potri.019G099900.1 | [AT4G17980.1](http://planttfdb.cbi.pku.edu.cn/tf.php?sp=Ath&did=AT4G17980.1) | regulation of transcription, DNA-templated |
| *NAC170* | Potri.T074200.1 | [AT4G12020.1](http://planttfdb.cbi.pku.edu.cn/tf.php?sp=Ath&did=AT4G12020.1) | regulation of transcription, DNA-templated; defense response; signal transduction |
